# Removal of anionic dye Congo red from aqueous environment using polyvinyl alcohol/sodium alginate/ZSM-5 zeolite membrane

**DOI:** 10.1038/s41598-020-72398-5

**Published:** 2020-09-22

**Authors:** Sabarish Radoor, Jasila Karayil, Jyotishkumar Parameswaranpillai, Suchart Siengchin

**Affiliations:** 1 Department of Mechanical and Process Engineering, The Sirindhorn International Thai-German Graduate School of Engineering (TGGS), King Mongkut’s University of Technology North Bangkok, 1518 Wongsawang Road, Bangsue, Bangkok, 10800 Thailand; 2 Government Women’s Polytechnic College, Calicut, Kerala India

**Keywords:** Environmental sciences, Chemistry, Materials science

## Abstract

In this study, a novel PVA/SA/ZSM-5 zeolite membrane with good regeneration capacity was successfully prepared by solvent casting technique. The properties of the membranes were assessed by employing different characterization techniques such as X-ray diffraction (XRD), Fourier-transform infrared spectroscopy (FT-IR), scanning electron microscope (SEM), optical microscopy (OP), thermogravimetric analysis (TGA), contact angle and universal testing machine (UTM). XRD, TGA and UTM results revealed that the crystallinity and thermo-mechanical performance of the membrane could be tuned with zeolite content. The successful incorporation of zeolite into the polymer matrix was confirmed by FT-IR, SEM and OP analysis. The adsorption ability of the as-prepared membrane was evaluated with a model anionic dye, Congo red. Adsorption studies show that the removal efficiency of the membrane could be tuned by varying zeolite content, initial concentration of dye, contact time, pH and temperature. Maximum dye adsorption (5.33 mg/g) was observed for 2.5 wt% zeolite loaded membrane, at an initial dye concentration of 10 ppm, pH 3 and temperature 30 °C. The antibacterial efficiency of the membrane against gram-positive (*Staphylococcus aureus*) and gram-negative bacteria (*Escherichia coli*) was also reported. The results show that membrane inhibits the growth of both gram-positive and gram-negative bacteria. The adsorption isotherm was studied using two models: Langmuir and Freundlich isotherm. The results show that the experimental data fitted well with Freundlich isotherm with a high correlation coefficient (R^2^ = 0.998). Meanwhile, the kinetic studies demonstrate that pseudo-second-order (R^2^ = 0.999) model describe the adsorption of Congo red onto PVA/SA/ZSM-5 zeolite membrane better than pseudo-first-order (R^2^ = 0.972) and intra particle diffusion model (R^2^ = 0.91). The experimental studies thus suggest that PVA/SA/ZSM-5 zeolite could be a promising candidate for the removal of Congo red from aqueous solution.

## Introduction

Majority of industries dumped untreated water directly into water bodies, thus contaminating it with toxic metals, dyes, pesticides and insecticides^1–3^. The severity of water pollution is scaling up steadily thus making it one of the major issues to be solved by the urban world. Water pollution is not only a threat to the environment but it also adversely affects humans and aquatic species^4,5^ It is estimated that 17–20% of industrial water pollution comes from dyeing industries^6^. Azo dye is the main class of synthetic dye which represents about 90% of all organic colorant^7^. Congo red (CR) is one of the best examples for azo dye and is chemically known as sodium salt of 3, 3′-([1,1′-biphenyl]-4,4′-diyl) bis (4-aminonaphthalene-1-sulfonic acid)^8^. It is widely used in wood, pigment, textile, dyeing, plastic and printing industries^9–13^. It is toxic in nature and pose a threat to both humans and animals. It causes several health issues in humans such as anorexia, weakness and gastrointestinal irritation^14,15^. As it is a benzidine based dye, it is suspected to have carcinogenic and mutagenic property^16,17^. Congo red enters into the water bodies through several industrial effluents. Owing to its high structural stability, it is quite resistant towards biodegradation and thus Congo red persist as contaminant in water environment for a long period of time. In this context, it is worthwhile to find a suitable adsorbent for the removal of Congo red from water^18^.

Water quality could be improved by various treatment methods such as flocculation, ion exchange, ozonation and adsorption^19–22^. Adsorption technique is one of the frequently adapted methods to improve the quality of water^23–25^. The interest in this technique comes from its ecofriendly and cost-effective nature, availability of wide range of adsorbent, simple experimental setup and regeneration of adsorbent^26^. Synthetic adsorbent exhibits high efficiency for the removal of toxic metals and dyes from aqueous medium. Therefore, it has been widely used to improve the quality of polluted water^27,28^. However, most of the synthetic adsorbents are toxic in nature and causes environmental pollution. Thus in recent years, there is great scientific interest in employing natural adsorbents such as clay, zeolite, date stones, rice husk and orange peel for dye removal studies^29–33^.

Polymers and polymer composites have been successfully used as adsorbent for water purification purpose. The properties such as good stability, reusability and selectivity, make them one of the promising candidates for water treatment purpose^34–36^^.^ Ample reports are available in literature regarding the use of polymer composite membranes for removing textile dyes from water^37–39^. For instance, Mahmoodi et al.^40^ developed PVA-chitosan nanofiber and reported its ability to remove anionic dye such as Direct Red and Reactive Red from water. Recently, Hu and coworker^41^ developed tannic acid/PVA/sodium alginate hydrogel bead which exhibit excellent adsorption power (adsorption capacity: 147.06 mg/g) for methylene blue. Kloster et al.^42^ reported that chitosan/iron oxide films could be a potential adsorbent for Congo red and possess sorption capacity of 700 mg/g. Kalantari et al.^42^ investigated the dye adsorption ability of chitosan/polyvinyl alcohol/talc composite and reported that the prepared composite displayed good adsorption efficiency for Congo red as well as methyl orange. Similarly, Habiba et al.^43^ successfully employed electro spun chitosan/polyvinyl alcohol/TiO_2_ nanofiber for the removal of Congo red and methyl orange from water. Zhao et al.^44^ proposed kaolin/calcium alginate membrane as a suitable adsorbent for the removal of toxic dye (brilliant blue G250, Congo red, and methylene blue). The membrane exhibits removal rate of 100%, 95.22%, and 62.86%, respectively for brilliant blue G250, Congo red, and methylene blue. Zhang et al.^45^ developed a novel chitosan/alginate sponge using freeze dry method and employed it for the removal of Congo red from aqueous medium. Based on the experimental result they report that 0.1 g of adsorbent has high removal efficiency (98.97%) for Congo red.

Zeolite is a naturally occurring aluminosilicate with wide range of application ranging from catalysis to water purification^46,47^. High surface area along with porous nature is responsible for its remarkable adsorption power^48,49^. The porosity of zeolite could be modified by several techniques such as templating method, dealumination, desilication etc.^50–52^. Zeolite containing both micro and mesopores are termed as hierarchical zeolites and are superior to conventional zeolite. Zeolites based polymer composites have been successfully employed for the removal of toxic contaminants from water^53,54^. However, there is no report on the usage of hierarchical zeolite-based polymer composite for the removal of Congo red from water. So, we thought it worthwhile to synthesis a hierarchical zeolite and employed it to fabricate an ecofriendly polymer membrane for Congo red removal. The polymer we choose for the current work is polyvinyl alcohol (PVA) and sodium alginate (SA), which are both ecofriendly and biocompatible in nature. The dye adsorption efficacy of the prepared membrane in terms of various experimental conditions such as zeolite loading, initial dye concentration, contact time, pH and temperature was investigated. The antibacterial activity and the reusability of the prepared membrane is also presented. The adsorption isotherm and kinetics of Congo red adsorption onto the membrane is also highlighted in this paper.

## Methods

### Materials

**Figure 1:**
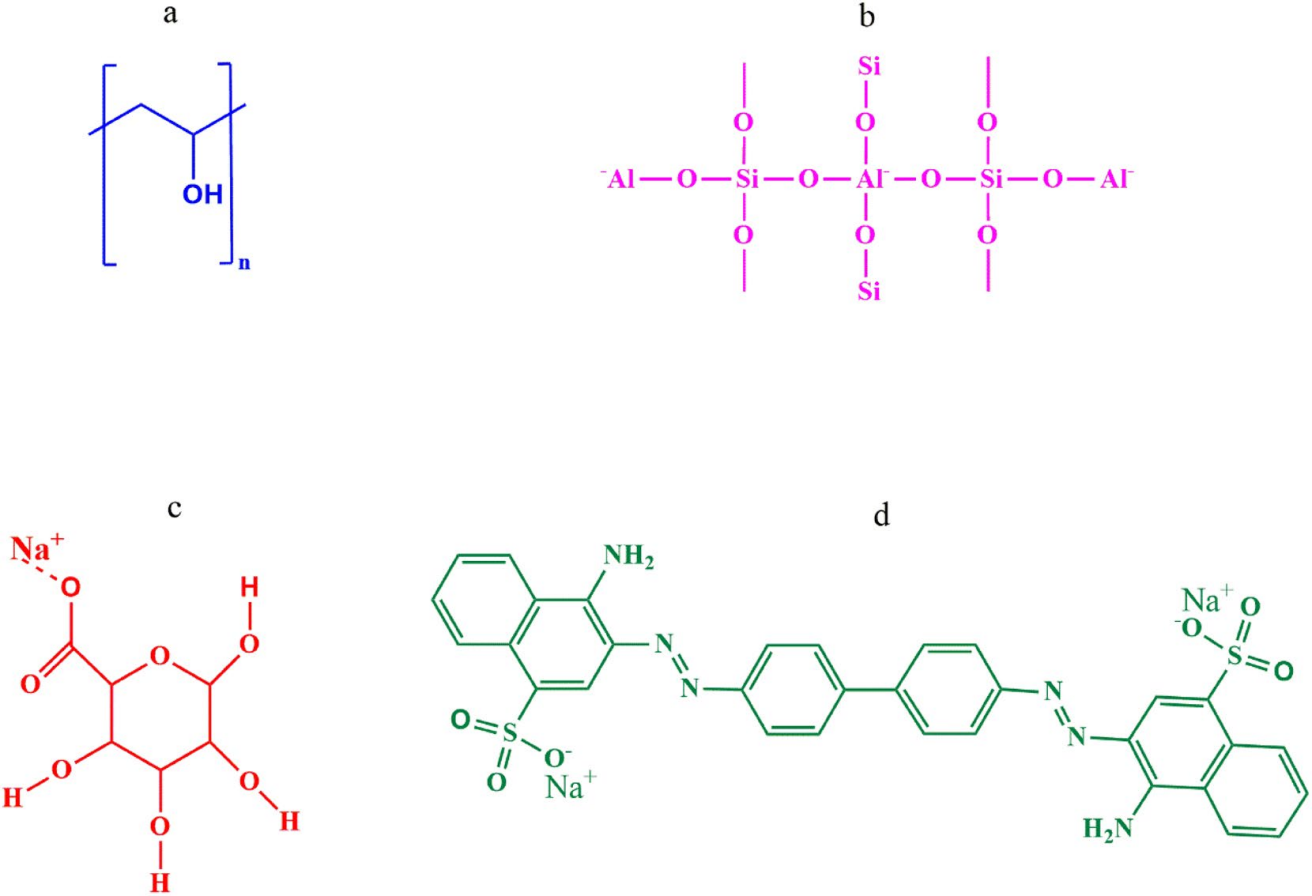
Molecular structures of: (**a**) PVA, (**b**) ZSM-5 zeolite, (**c**), Sodium alginate (**d**) Congo red dye.

The polymers polyvinyl alcohol (C_2_H_4_O, Mw = 130,000: hydrolysis degree: 99%) and anionic dye, Congo red ((C_32_H_22_N_6_Na_2_O_6_S_2_); purity: 99.0%) was purchased from Ajax Finechem Pvt. Ltd. (Thailand). The crosslinking agent glutaraldehyde (C_5_H_8_O_2_) were procured from Loba Chemie Products Limited. (Thailand). Sodium alginate ((C_6_H_7_NaO_6_)_n_, average Mw = 60,000) were obtained from Nice chemical Pvt. Ltd. (India). Tetra propyl ammonium hydroxide ((CH_3_CH_2_CH_2_)_4_NOH, TPAOH), tetraethyl orthosilicate (C_8_H_20_O_4_Si, TEOS) and aluminum isopropoxide (C_9_H_21_AlO_3_, AIP) were procured from Sigma Aldrich Co. Ltd (India). Soluble starch (C_6_H_10_O_5_)_n_ which was used as templating agent were of reagent grade and was procured from Merck (India). All the chemicals used in the study were of analytical grade and were used as received without further purification. The polymer solution and dye solution were prepared in distilled water. Figure 1 depicts the chemical structure of the materials used in the study.

### Fabrication of hierarchical ZSM-5 zeolite

The procedure for the synthesis of hierarchical ZSM-5 zeolite is briefly described below. To the properly mixed tetra propylammonium hydroxide (TPAOH; 2.11 g) and aluminum isopropoxide (AIP; 0.03 g) solution, tetraethyl orthosilicate (TEOS; 3.46 g) solution was added dropwise with constant stirring for 5 h at room temperature. This was followed by the addition of meso templating agent, starch (0.2 g) and the mixture was magnetically stirred for 2 h to attain homogeneity. The resulting mixture was then concentrated in a rotavapor at 80 °C for 30 min until a viscous solution is obtained, which was transferred into a Teflon‐lined stainless-steel autoclave, and kept at 80 °C for 24 h and crystallized at 175 °C for 6 h. The solid product was collected, washed with deionized water, dried in air, and calcined at 550 °C for 5 h in a muffle furnace to remove the starch and organic structure directing agents^55,56^.

### Fabrication of PVA/sodium alginate/ZSM-5 zeolite membrane

PVA/sodium alginate/ZSM-5 zeolite membrane was fabricated by solvent casting method. At first, required amount of polyvinyl alcohol and sodium alginate were dissolved separately in distilled water under constant stirring to obtain 9 and 1.5 wt% of PVA and SA solution respectively. The resultant solution was then mixed and magnetically stirred for 3 h. Afterwards, hierarchical ZSM-5 zeolite of varying concentration ranging from 0.5 to 2.5 wt% were added and stirred at room temperature for 2 h to attain homogeneity. Once the solution was properly homogenized, 0.1 mL of cross-linker glutaraldehyde (GA) in acidic medium (HCl) were added in dropwise. The blended solution was casted on a petri dish and kept for air drying for a period of 5 days. The membrane was later peeled out from the petri dish and used for adsorption studies. The membranes containing 0, 0.5, 1.5 and 2.5 wt% of ZSM-5 zeolite is designated as PSZ-0, PSZ-1, PSZ-2, and PSZ-3 respectively. Chemical compositions of membranes are displayed in Table 1.

**Table 1:** The composition of the casting solution.

Sample code	PVA (wt%)	SA (wt%)	ZSM-5 (wt%)
PSZ-0	9	1.5	0
PSZ-1	9	1.5	0.5
PSZ-2	9	1.5	1.5
PSZ-3	9	1.5	2.5

### Characterisation

The crystallinity of the PVA/SA/ZSM-5 zeolite membrane was identified by Rigaku miniflex 600 benchtop X-ray diffractometer with Cu-Kα radiation as X-ray source. The XRD spectrum was recorded from 5 to 50° (2θ) at a scan rate of 2°/min. The chemical structure of the membrane was monitored by using Jasco 4,700 FT-IR spectrometer in the range of 450–4,000 cm^−1^ with 4 cm^−1^ resolution. A thermogravimetric analyser Mettler Toledo TGA/DSC 3 + HT/1,600 instrument was employed to investigate the thermal stability of membranes. The analysis was carried out in the temperature range of 50–750 °C at a heating rate of 10 °C/min in N_2_ atmosphere. The surface morphology of the membranes was assessed by scanning electron microscope (FEI Quanta 450) and optical microscopy (Olympus BX43 series) instrument. Universal testing machine (QC-506M1-204) was employed to investigate the mechanical properties of the membranes. Static water contact angle of the membranes was probed using Digidrop goniometer. Adsorption experiments were conducted using UV–visible spectrometer Specord (UV-210) at a characteristic wavelength of 492 nm.

### Adsorption studies

For adsorption experiments, 0.075 g of membrane was added to 40 mL of Congo red solution and stirred at room temperature. After regular time interval the dye solution was withdrawn, filtered and analysed using UV–visible spectrophotometer at a characteristic wavelength of 492nm. The concentration of Congo red at different time interval was determined from the calibration graph. The percentage dye removal efficiency (R) and equilibrium adsorption capacity (q_e_) of the membrane were calculated using the following equations:

1 $$ {\text{q}}_{{\text{e}}} = \frac{{({\text{C}}_{0} - {\text{C}}_{{\text{e}}} ){\text{V}}}}{{\text{W}}} $$

2 $$ {\text{R}} = \frac{{{\text{C}}_{0} - {\text{C}}_{{\text{e}}} }}{{{\text{C}}_{0} }} \times 100\left( \% \right) $$

Here C_0_ and C_e_ (mg/L) represent the initial and equilibrium concentrations of Congo red respectively; V (L) is the volume of Congo red solution and W(g) is the weight of the dried membrane^30^.

### Antibacterial studies

The disk diffusion agar method was employed to assess the antimicrobial property of the PVA/SA/ZSM-5 zeolite membranes. The culture medium was prepared using Muller-Hinton agar seeded with 100 µL of bacterial strain (*Escherichia coli* and *Staphylococcus aureus*). Membranes were cut in a circular shape with 1 cm diameter and placed on this agar plates, which was later incubated at 37 °C for 24 h. The diameter of the inhibition zone formed around the specimens was measured and photographed^57,58^.

### Reusability

For recyclability test, 0.075 g of Congo red loaded membrane is immersed in 0.1 N of HCl under constant stirring for 3 h. The membrane was then separated from acid solution, washed several times with distilled water, dried and reuse for further adsorption process^59^.

## Result and discussion

**Figure 2:**
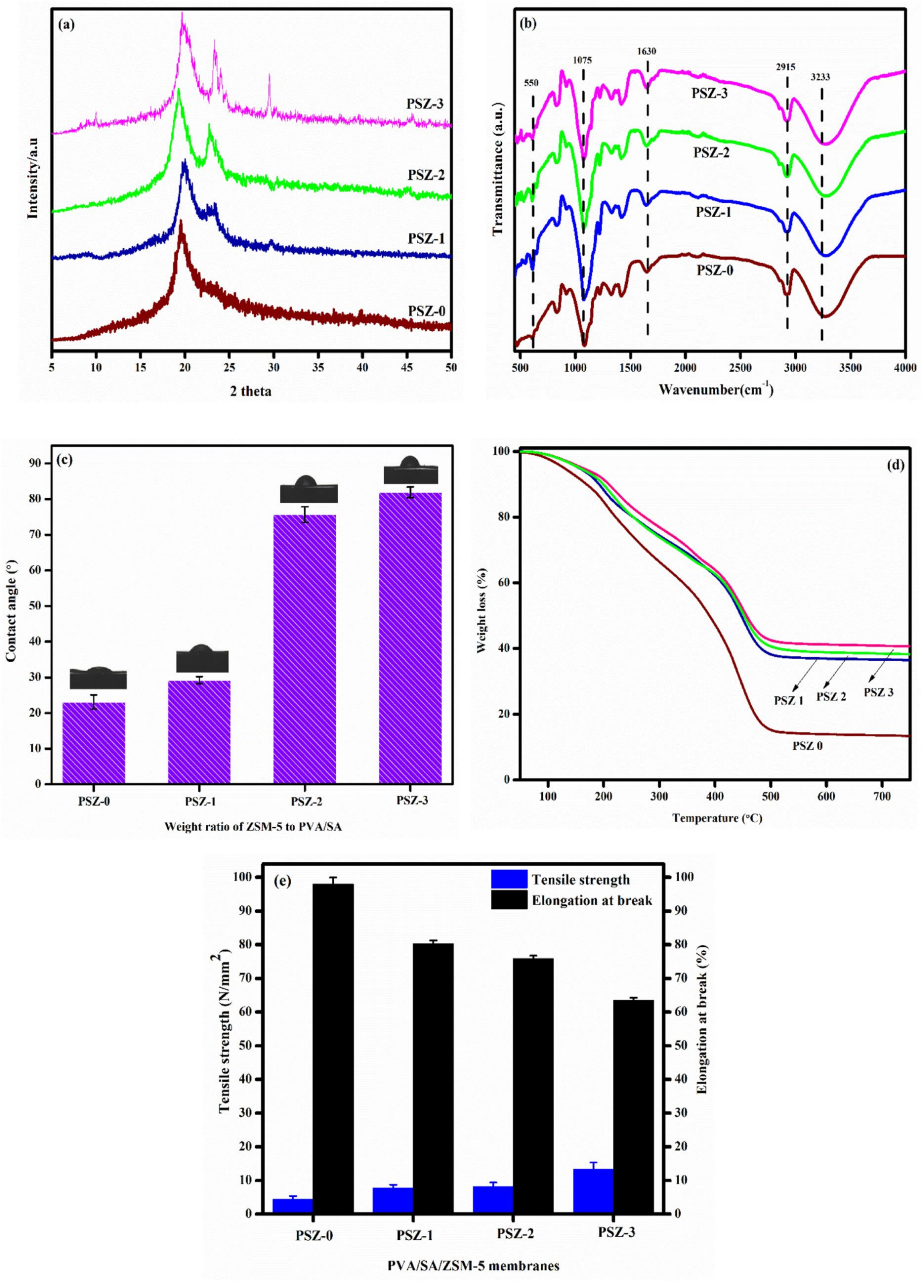
(**a**) XRD, (**b**) FTIR (**c**) contact angle (**d**) TGA and (**e**) mechanical properties of PVA/SA/ZSM-5 zeolite membranes.

Polymer with good crystallinity tends to improve the thermal and mechanical stability of the membrane. This prompted us to evaluate the effect of zeolite on the crystalline nature of PVA/SA membrane. The XRD pattern of PVA/SA/ZSM-5 zeolite membrane is shown in Fig. 2a. The crystalline peak centered at 2θ = 20°, is attributed to the reflection from (100) and (200) plane of PVA and SA^60,61^. Comparison of XRD pattern of pristine PVA/SA membrane with PVA/SA/ZSM-5 zeolite membranes shows that both the membrane possesses similar diffraction pattern. However, with increases in zeolite concentration the intensity of peak at 2θ = 22–25° increases; attributed to the characteristic MFI peak of zeolite^62^. The XRD result thus shows that zeolite retains the overall crystallinity of the membrane. FTIR is one of the powerful tools to identify the functional groups and to understand the chemical interaction between molecules. FTIR spectrum of PVA/SA/ZSM-5 zeolite membranes are illustrated in Fig. 2b. The peak observed at 3,233 cm^−1^ is assigned to the hydroxyl stretching band of PVA, sodium alginate and zeolite^63^. The alkyl stretching present in PVA, sodium alginate and zeolite can be seen at 2,915 cm^−1^ and the sharp peak centered at 1,630 cm^−1^ is assigned to the characteristic C=O stretching of carboxyl group of sodium alginate^64^. A prominent band at 1,075 cm^−1^ can be seen in the FTIR spectra and is attributed to the acetal bridge (C–O–C) present in the crosslinked structure of PVA and GA. In the case of zeolite loaded membrane, we can observe a new peak at 550 cm^−1^, assigned as the characteristic structure of zeolite^30^. The intensity of this characteristics peak is found to increase with zeolite content, thus confirming successful incorporation of zeolite in PVA/SA membrane. A prominent blue shift observed in the characteristic peak of zeolite indicates the formation of hydrogen bond between PVA and zeolite (Fig (S1)). Contact angle measurement is an important analysis which is used to elucidate the hydrophilicity or wettability of the membrane. Perhaps, contact angle and hydrophilicity is inversely related to each other. Membrane with low contact angle is said to possess high hydrophilic character. On the other hand, a high value of contact angle implies greater hydrophobicity. The contact angle measurement of PVA/SA/ZSM-5 zeolite membrane is presented in Fig. 2c. It can be seen from Fig. 2c that the PVA/SA membrane possess low contact angle (23.5°). This could be due to the presence of hydrophilic functional groups such as –OH and –COOH. However, the contact angle of PVA/SA/ZSM-5 zeolite membrane is relatively higher than pristine membrane and its value is found to escalates from 23.5° to 81.93° with zeolite loading. This implies that PVA/SA/ZSM-5 zeolite membrane is less hydrophilic than pristine membrane. Therefore, the water drop placed on the surface of zeolite loaded membrane becomes more spherical (Fig. 2c) with increasing zeolite content. This is agreement with the previous report^65,66^. The thermal behavior of the membrane was examined using TGA analysis and the result is presented in Fig. 2d. It can be noticed that the membranes exhibit weight loss in three stages. The initial weight loss occurred in the temperature range 50–150 °C corresponds to the removal of loosely bound moisture in the membrane. The second stage of degradation around 170 to 310 °C is due to decomposition of PVA and sodium alginate. Final stage observed around 330–520 °C corresponds to degradation of zeolite crystals^2,67^. Wang et al.^68^ noted a similar degradation temperature (100, 200 and > 350 °C) for PVA/SA/ZSM-5 zeolite membrane and is ascribed to the removal of water, PVA, SA and zeolite from the membrane framework. It is evident from Fig. 2d that weight loss and the slope of degradation gets gradually reduced from membrane PSZ-0 to PSZ-3 (84.82% to 58.37%). This is expected owing to the high zeolite content which increases the thermal stability of the membrane. Mechanical strength and mechanical stability are important factor for membrane studies. Earlier report shows that addition of nanoparticles such as zeolites, clay, laponite will improve the polymer stability^69,70^. The mechanical properties of different membrane were shown in Fig. 2e. An improvement in the tensile strength of the membrane with zeolite percentage was noted. This is attributed to the enhancement of chemical interaction between the zeolite and polymer matrix. However, the elongation at break decrease with zeolite content. Prasad et al. also observed a similar type of behavior for PVA/SA/ZSM-5 zeolite system^71^.

**Figure 3:**
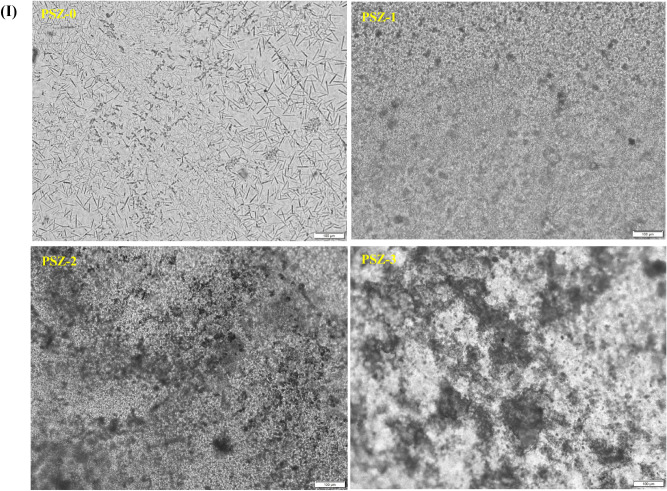

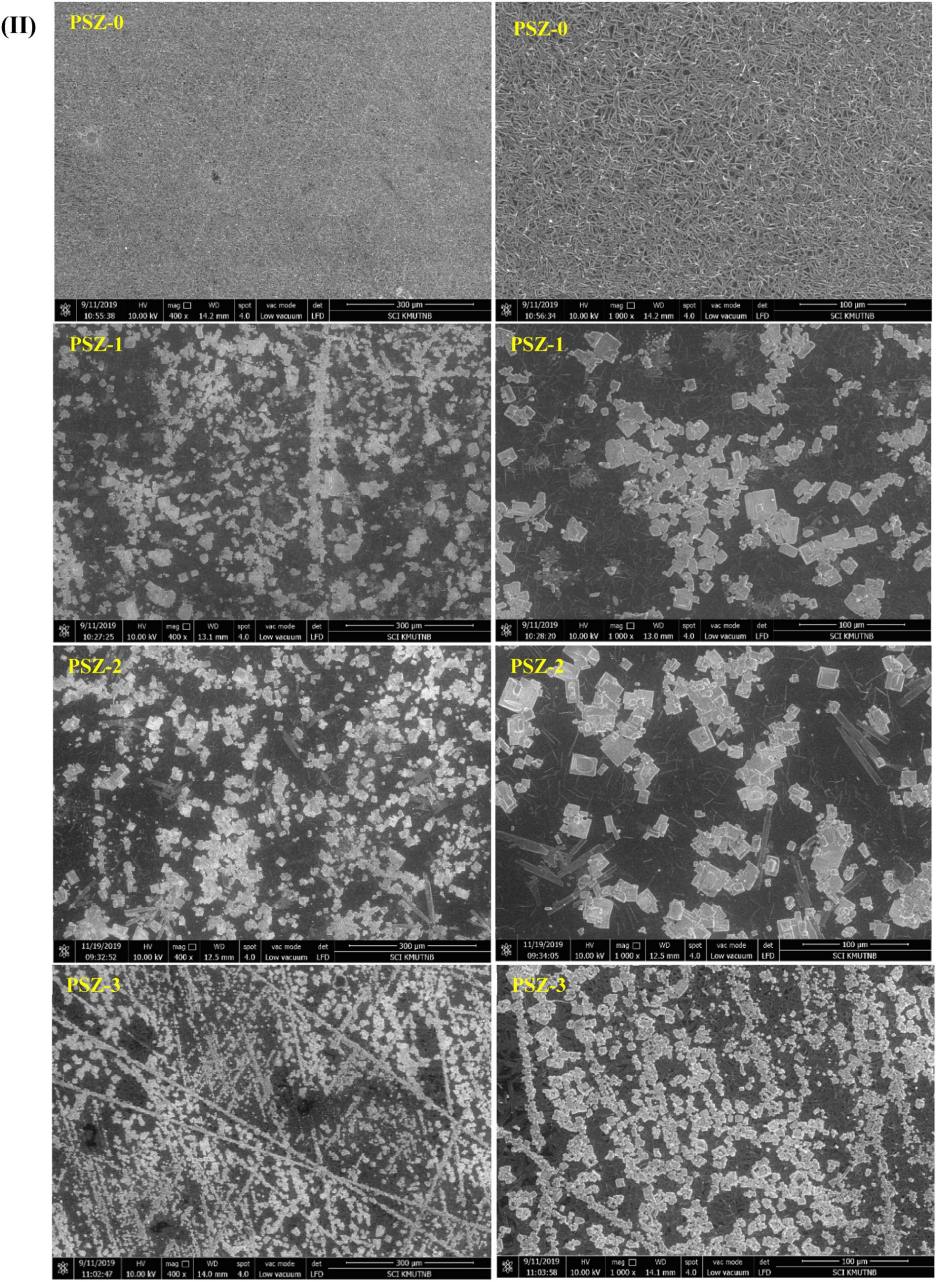
(**I**) Optical microscopic and (**II**) SEM image of PVA/SA/ZSM-5 zeolite membranes with low and high magnifications.

The effect of zeolite on the surface morphology of the pristine PVA/SA membrane was evaluated using SEM and optical microscopic technique. The surface morphology of pristine PVA/SA and PVA/SA membrane loaded with different percentage of zeolite (PSZ-1 to PSZ-3) is presented in Fig. 3(I,II). It can be seen from Fig. 3 that PVA/SA possesses a rod like morphology. On the other hand, PVA/SA/ZSM-5 zeolite membranes displayed a large number of cubic particles on the surface of polymer matrix; attributed to the presence of zeolite. With increase in zeolite percentage, the distribution of these particles is found to increase and at high concentration, a slight agglomeration can be noted. The SEM analysis thus proved that zeolite particle is successfully incorporated in PVA/SA membrane. Optical microscopic images were in complimentary to SEM analysis and supports the distribution of zeolite in the polymer matrix (Fig. 3(II)).

### Adsorption studies

**Figure 4:**
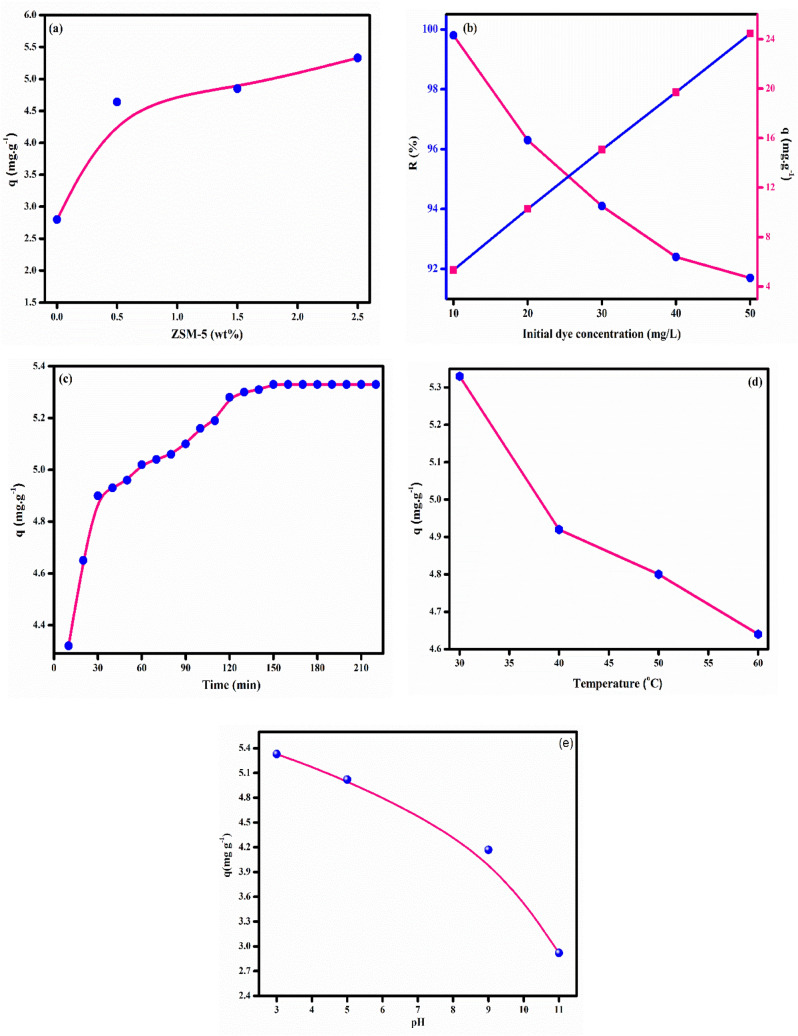
Effect of different parameters (**a**) zeolite dosage, (**b**) initial dye concentration, (**c**) contact time, (**d**) temperature, (**e**) pH on adsorption of Congo red onto PVA/SA/ZSM-5 zeolite membrane (adsorbent dosage = 2.5 wt%, initial CR concentration = 10 ppm, contact time = 130 min, pH = 3 and temperature = 30 °C).

The adsorption ability of the prepared membranes was tested using a model anion dye, Congo red. The effect of operation parameters such as zeolite loading, initial dye concentration, contact time, pH and temperature on the adsorption behavior of the membranes was investigated in detail. Zeolite is one of the versatile materials with high surface area and has been widely used for the adsorption of dyes, toxic metal and gases^43^. Our previous reports show that it is an excellent adsorbent for the removal of methylene blue (MB) and methyl orange (MO) from aqueous solution^54,72^. In order to optimize the adsorbent dosage, we have varied the zeolite dosage from 0.5 to 2.5 wt% and investigated its effect on the adsorption capacity of the membrane. It can be deduced from Fig. 4a that with increase in zeolite dosage, the adsorption capacity increases and the membrane loaded with 2.5 wt% of zeolite exhibits maximum adsorption capacity (5.33 mg/g). The linear increase in the adsorption capacity with adsorbent dosage is in agreement with the previous reported work and could be explained on the basis of availability of vacant active site on the membrane^73^. With increase in zeolite content, the number of active site and surface area of the membrane increases. Thus, zeolite provides a favorable environment for the adsorption of Congo red onto the membrane. Our observation agrees well with the work of Briao et al.^74^ The authors noticed a significant enhancement in the adsorption capacity of chitin/zeolite membrane for crystal violet with zeolite dosage. Initial dye concentration plays a significant role in dye adsorption process. The effect of initial dye concentration on the adsorption capacity and percentage dye removal was investigated by varying initial dye concentration from 10 to 50 ppm and the results are presented in Fig. 4b. It is evident from Fig. 4b that with increase in C_o_, the q_e_ increases from 5.33 to 24.45 mg/g, while the removal efficiency decreases from 99.9 to 90%. Our result is in consistent with the previous reports which stated that initial dye concentration renders necessary driving force to reduce the resistance of mass transfer from liquid to solid phase^75^. Consequently, the collision between dye and the adsorbent increases and the adsorption process become more favorable. Baheri et al.^76^ reported that that adsorption capacity of PVA/4A zeolite membrane for methylene blue (MB) dye rises from 4.90 to 41.08 mg/g on increasing the initial dye concentration from 5 to 100 mg/g. The decreases in dye removal percentage with initial concentration of Congo red dye is attributed to the saturation of active site under high concentration condition of Congo red^77^. Contact time is an important parameter in dye adsorption studies. The effect of contact time on the Congo red removal is depicted in Fig. 4c. It can be noted that during the initial period the adsorption capacity shoots up sharply and after 2.5 h it almost attain equilibrium. This is probably due to the availability of large number of active sites in the initial phase of the adsorption process which facilitates the binding of Congo red onto the membrane. However, with passage of time as more and more dye molecules get adsorbed on the surface of the membrane the adsorption process become less favoured. Eventually, all active sites get occupied by dye molecules and the adsorption capacity remains almost constant^78^. Chen et al.^79^ reported that on increasing the contact time from 20 to 180 min, Congo red adsorption capacity of PDA@DCA-COOH membrane enhances from 30 mg/g to 79.33 mg/g. After 180 min, no further improvement in the adsorption capacity was noted and thus the authors selected 180 min for performing the adsorption studies. Solution temperature is reported to influence the adsorption behavior of the membrane. The variation of temperature on the dye uptake ability of PVA/SA/ZSM-5 zeolite membrane is shown in Fig. 4d. It can be noted that the adsorption capacity drops from 5.33 to 4.62 mg/g on increasing the solution temperature from 30 to 60 °C. The negative effect of temperature on the adsorption capacity could be correlated with previous reported work and implies that Congo red adsorption onto the membrane is exothermic in nature^80^. Another plausible reason could be the weakening of electrostatic interaction between dye and the membrane with temperature. Thus, we can conclude that high temperature is unfavorable for the adsorption of Congo red by PVA/SA/ZSM-5 zeolite membrane. Solution pH play a crucial role in dye adsorption studies, especially if the mechanism of adsorption is electrostatic in nature. A change in solution pH will alter the properties of both dye as well as the adsorbent. Therefore, optimizing solution pH is a necessary requirement for adsorption process. The role of pH on the Congo red adsorption by PVA/SA/ZSM-5 zeolite membrane was studied by varying the pH from 3 to 11. It can be seen from Fig. 4e that with increase in pH, the adsorption capacity of the membrane decreases from 5.33 to 2.92 mg/g. This could be due to difference in the surface charge of the membrane with pH. At low pH, the surface of membrane gets protonated and thus attracts anionic Congo red^81^. However, in basic pH the membrane surface tends to become negative and repel the anionic Congo red. In addition to this, high pH increases the competition between Congo red dye and hydroxide (OHˉ) for the same adsorption site.

### Adsorption isotherm

Knowledge of adsorption isotherm is essential to design the adsorption system, as it shed light on adsorbate-adsorbent interaction and adsorption capacity of the adsorbent. Freundlich, Langmuir, Temkin and Dubinin-Radushkevich isotherm models are commonly used to understand the adsorption process^79,81^. In the present work, the adsorption behavior of the prepared membrane was evaluated using Freundlich and Langmuir isotherm models.

### Langmuir model

According to Langmuir model, the surface of adsorbent is homogenous and possesses finite number of energetically equivalent adsorption sites. This model thus suggests a uniform adsorption of adsorbate on the surface of adsorbent and neglects any interaction between the adsorbed molecules^78^. Langmuir model is given by the following expression.

3 $$ {\text{q}}_{{\text{e}}} = \frac{{{\text{q}}_{{\text{m}}} {\text{K}}_{{\text{L}}} {\text{C}}_{{\text{e}}} }}{{1 + {\text{K}}_{{\text{L}}} {\text{C}}_{{\text{e}}} }} $$

Here, q_e_ corresponds to the equilibrium amount of adsorbate or adsorption capacity (mg/g), while C_e_ represents equilibrium concentration of adsorbate (mg/L). K_L_ and q_m_ represent Langmuir constant and is related to adsorption energy and adsorption capacity respectively. The linear form of Langmuir isotherm model is expressed as follows

4 $$ \frac{{{\text{C}}_{{\text{e}}} }}{{{\text{q}}_{{\text{e}}} }} = \left( {\frac{{{\text{C}}_{{\text{e}}} }}{{{\text{q}}_{{\text{m}}} }}} \right) + \left( {\frac{1}{{{\text{K}}_{{\text{L}}} *{\text{q}}_{{\text{m}}} }}} \right) $$

The plot of C_e_/q_e_ vs C_e_ results in a straight line with q_m_ as slope and K_L_ as intercept. Another important parameter obtained from Langmuir model is Langmuir separation factor, R_L_. This dimensionless constant can be used to predict the feasibility and favorability of the adsorption process. The adsorption is said to be favorable if R_L_ is between 0 and 1, irreversible if R_L_ = 0, linear if R_L_ = 1 and unfavorable if R_L_ > 1. R_L_ is related to K_L_ and C_o_ by the following expression.

5 $$ {\text{R}}_{{\text{L}}} = \frac{1}{{1 + {\text{K}}_{{\text{L}}} {\text{C}}_{0} }}$$

### Freundlich model

This model assumes non-uniform distribution of adsorbate on the surface of adsorbent and describes a multilayer adsorption. The non-linear form of Freundlich isotherm is given below;

6 $$ {\text{q}}_{{\text{e}}} = {\text{K}}_{{\text{F}}} {\text{C}}_{{{\text{eq}}}}^{{1/{\text{n}}}}  $$

where q_e_ is the amount of Congo red adsorbed onto the membrane at equilibrium (mg/g) and C_e_ is the equilibrium concentration of Congo red in solution (mg/L). K_F_ and 1/*n* are adsorption capacity and adsorption intensity (surface homogeneity) which is obtained from the slope and intercept of the plot between ln q_e_ and ln C_e_ respectively. The value of 1/*n* is indicative of favorability of the adsorption. For instance, 1/*n* value between 0 and 1 implies favorable adsorption while 1/*n* > 0 and equal to zero is considered as irreversible and unfavorable adsorption respectively^82^.

The linear form of Freundlich isotherm is represented as follows;

7 $$ {\text{ln}}\left( {{\text{q}}_{{\text{e}}} } \right) = {\text{InK}}_{{\text{F}}} + \frac{1}{{\text{n}}}*{\text{In}}({\text{C}}_{{\text{e}}} ) $$

**Figure 5:**
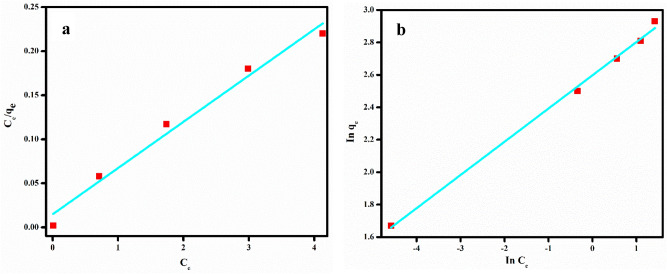
(**a**) Langmuir and (**b**) Freundlich isotherm plot for the adsorption of Congo red onto PVA/SA/ZSM-5 zeolite membrane (adsorbent dosage = 2.5 wt%, initial CR concentration = 10 ppm, contact time = 130 min, pH = 3 and temperature = 30 °C).

The applicability of these two models to explain the Congo red adsorption onto PVA/SA/ZSM-5 zeolite membrane was verified by fitting the experimental adsorption data into the models (Fig. 5a,b) and the characteristic isotherm parameters derived from the isotherm models are presented in Table 2. It is evident from Fig. 5 that the experimental adsorption data fitted well with Freundlich model with high correlation ratio, (R^2^: 0.9985). Also, there is good agreement between calculated (4.8 mg/g) and experimental q_e_ value (5.33 mg/g) thus suggesting that the Freundlich isotherm model is more suitable to describe the adsorption of Congo red onto PVA/SA/ZSM-5 zeolite membrane and involves a multilayer adsorption process. The value of Freundlich constant, *n* was found to be 4.4 (Table 2), implying the favourable nature of adsorption process^83^.

**Table 2:** Adsorption isotherm parameters of Congo red onto PVA/SA/ZSM-5 zeolite membrane.

Exp	Langmuir			Freundlich		
q_e_, exp (mg/g)	q_m_ (mg/g)	K_L_	R^2^	K_F_ (mg/g)	n	R^2^
5.33	19	3.54	0.99	4.8	4.4	0.998

### Adsorption kinetics

Adsorption kinetics gives an idea about the controlling mechanism of adsorption process (chemical reaction, diffusion and mass transfer process) and provides information regarding adsorption rate and adsorbate residue time. To investigate the kinetics of Congo red adsorption on the PVA/SA/ZSM-5 zeolite membrane pseudo-first-order (PFO), pseudo-second-order (PSO) and intra-particle diffusion model were employed. The linear form of PFO and PSO is given below.

PFO model

8 $$ {\text{log}}\left( {{\text{q}}_{{\text{e}}} - {\text{q}}_{{\text{t}}} } \right) = {\text{logq}}_{{\text{e}}} - \frac{{{\text{K}}_{1} {\text{t}}}}{{2.303}} $$

PSO model

9 $$ \frac{{\text{t}}}{{{\text{q}}_{{\text{e}}} }} = \frac{1}{{{\text{K}}_{2} {\text{q}}_{{\text{e}}}^{2} }} + \frac{{\text{t}}}{{{\text{q}}_{{\text{e}}} }} $$

where K_1_ (min^−1^) is the pseudo-first-order rate constant, K_2_ (g/mg min^−1^) is the pseudo-second-order rate constant, q_t_ (mg/g) is the adsorption capacity at time t (min) and q_e_ (mg/g) is the adsorption capacity at equilibrium respectively, which is determined from the slope of log (q_e_ − q_t_) versus time and t/qt versus time of linear plot.

According to intra-particle diffusion model, the kinetics of adsorption involves diffusion of adsorbent into the pores of adsorbent and is represented as

$$ {\text{q}}_{{\text{t}}} = {\text{K}}_{{{\text{id}}}} {\text{t}}_{{}}^{{1/2}} $$

where K_id_ and C are intraparticle constants and is estimated from the slope and intercept of the plot between q_t_ vs t^1^^/2^.

**Figure 6:**
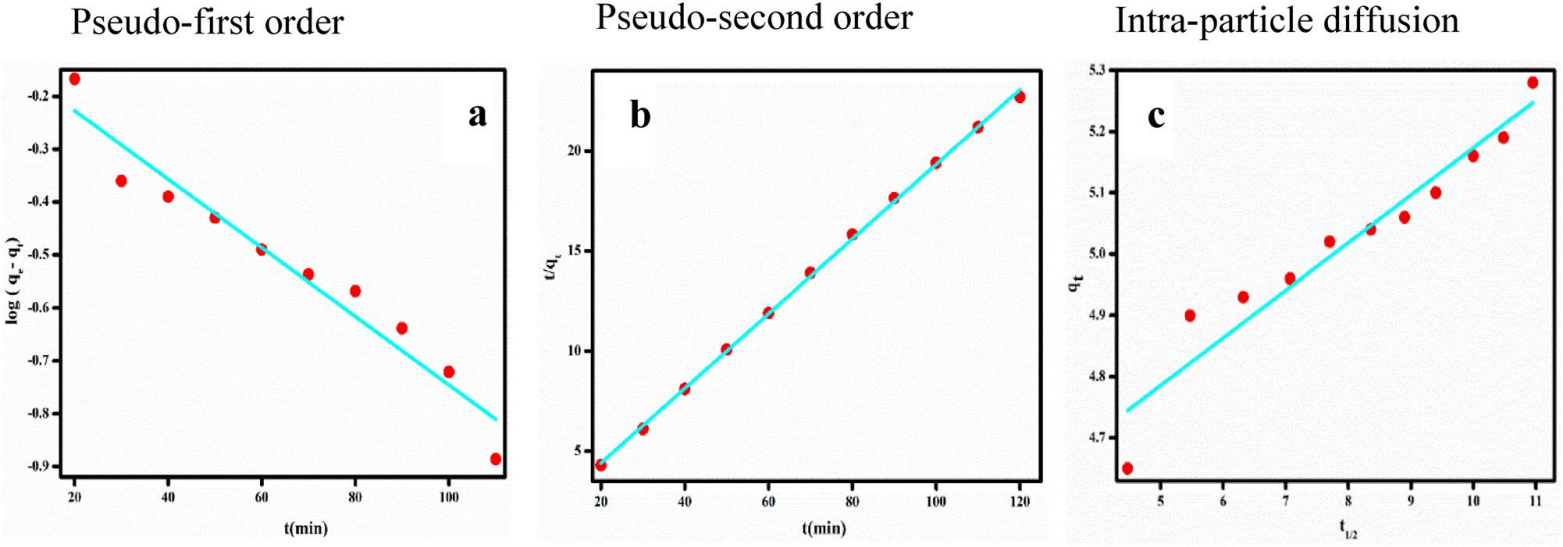
Plots of the pseudo-first, pseudo-second and intra-particle diffusion model for Congo red adsorption onto PVA/SA/ZSM-5 zeolite membrane (adsorbent dosage = 2.5 wt%, initial CR concentration = 10 ppm, contact time = 130 min, pH = 3 and temperature = 30 °C).

The kinetics of Congo red adsorption onto PVA/SA/ZSM-5 zeolite membrane was assessed by fitting the experimental kinetic data into the linearized form of PFO, PSO and intraparticle diffusion models (Fig. 6). The kinetic parameters obtained from the result are displayed in Table 3. It is evident from figure that our data fitted well in PSO model (R^2^-0.999) and the experimental value was very much close to the q_e_ value predicted using PSO model. This implies the suitability of the PSO model for describing the kinetic mechanism of Congo red adsorption on PVA/SA/ZSM-5 zeolite membrane.

**Table 3:** Kinetic parameter values for the Congo red adsorption on zeolite loaded PVA/SA membrane.

Exp	Pseudo-first-order			Pseudo-second-order			Intra-particle diffusion		
q_e_, exp (mg g^−1^)	K_1_ (min^−1^)	q_e_ (mg g^−1^)	R^2^	K_2_ (g mg^-1^ min^−1^)	q_e_ (mg g^−1^)	R^2^	K_id_ (g mg^-1^ min^−1/2^)	C (mg g^−1^)	R^2^
5.33	0.0147	1.1	0.972	0.0508	5.36	0.999	0.077	4.39	0.91

**Figure 7:**
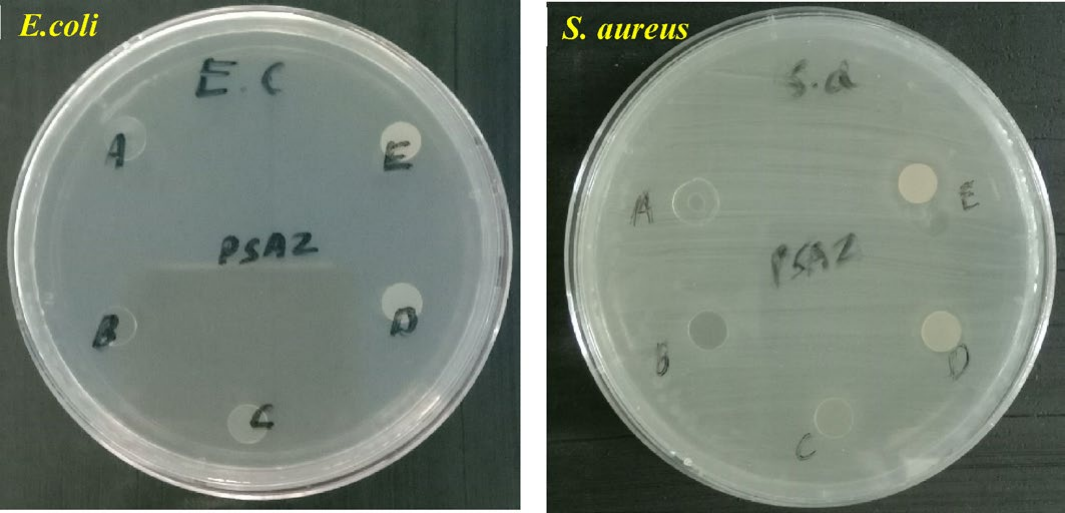
Photograph of antimicrobial test of different percentage of zeolite loaded on PVA/SA membrane against gram-negative and gram-positive: (**A**) PVA, (**B**) PSZ-0, (**C**) PSZ-1, (**D**) PSZ-2, (**E**) PSZ-3.

An adsorbent with good antibacterial property is beneficial for water treatment application. This prompted us to investigate the antibacterial property of the prepared membrane. The antimicrobial activity of pure PVA, PVA/SA and zeolite loaded PVA/SA was tested against gram-negative (*E. coli*) and gram-positive (*S. aureus*) by inhibition zone method (Fig. 7). Absence of inhibition zones surrounding the PVA/SA membrane, suggest that pristine PVA/SA membrane have no antibacterial property. One can observe that zeolite loaded membrane displayed significant inhibition action against gram-positive (*S. aureus*) and gram negative bacteria (*E. coli*). The diameter of inhibition zone is found to increase with zeolite content thus indicating that the antibacterial property of the PVA/SA membrane could be improved by incorporating ZSM-5 zeolite. The mechanism of action of zeolite on gram-positive bacteria remains uncertain. But, we presume that negatively charged zeolite could easily bind with the surface of gram-positive bacteria and causes the disruption of cell wall. This perhaps leads to the leakage of cellular material and leads to bacterial death. The weak inhibition action of zeolite against gram-negative bacteria could be due to the repulsion between zeolite and negatively charged surface of gram-negative bacteria.

**Figure 8:**
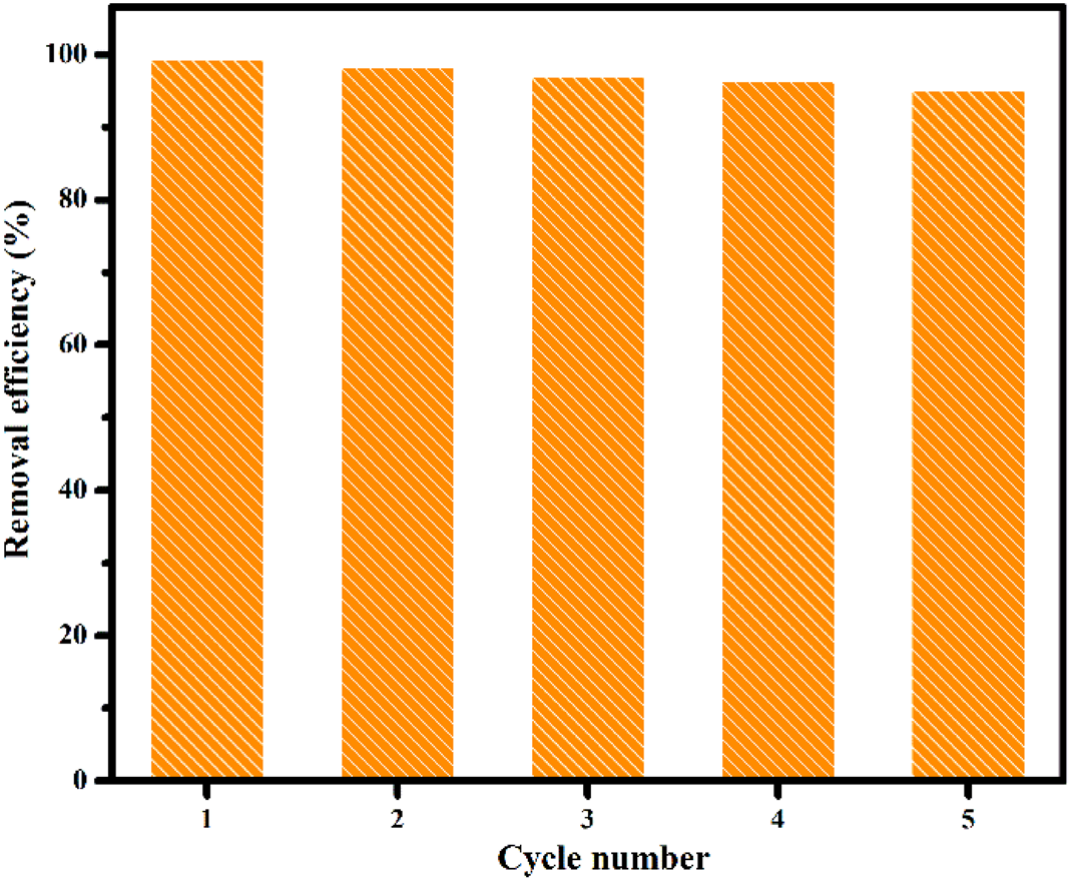
Recyclability test of Congo red adsorption on PVA/SA/ZSM-5 zeolite membrane.

Reusability test is generally employed to access the practicability of the membrane. So, in the present study we subjected the membrane to five desorption-adsorption cycle and the results is presented in Fig. 8. It is evident from the Fig. 8 that the membrane possesses good regeneration capacity even after 5 cycle. The small decrease in the removal efficiency while going from first (99.3%) to firth cycle (95.1%) could be due to the incomplete desorption of Congo red from the membrane^84^.

## Conclusion

In this work, we have successfully developed an ecofriendly adsorbent for the removal of toxic anionic dye, Congo red from aqueous medium. Based on different characterization technique’s such as XRD, FTIR, TGA and contact angle we found that the membrane is stable and the properties of the membrane such as crystallinity, thermal stability and hydrophilicity could be tuned by varying zeolite content. SEM and optical analysis revealed that the zeolite particle is successfully incorporated in the polymer matrix. The membrane was tested for Congo red removal from water. The optimized condition for the removal of Congo red from membrane is found to be as follows: zeolite content = 2.5 wt%; initial CR concentration = 10 ppm, contact time = 130 min, temperature = 30 °C and pH = 3. The adsorption isotherm and kinetics analysis revealed that the adsorption can be best described by Freundlich and PSO model respectively. The prepared membrane also possesses prominent antibacterial property and recyclability. Thus, from the current work, we can conclude that PVA/SA/ZSM-5 zeolite membrane could be a promising candidate for the removal of Congo red from polluted water.

## Supplementary information

Supplementary Information.
