# Supplementary material for: Removal of anionic dye Congo red from aqueous environment using polyvinyl alcohol/sodium alginate/ZSM-5 zeolite membrane
Source: Sci Rep. 2020 Sep 22;10:15452. doi: 10.1038/s41598-020-72398-5 (PMC7509836; doi:10.1038/s41598-020-72398-5)
Supplement: Supplementary file 1 — Supplementary Information. [file 41598_2020_72398_MOESM1_ESM.docx]

**Removal of anionic dye Congo red from aqueous environment using polyvinyl alcohol/sodium alginate/ZSM-5 zeolite membrane**

**Graphical abstract**

Sabarish Radoor^1*^, Jasila Karayil^2^, Jyotishkumar Parameswaranpillai^1^, Suchart Siengchin^1*^

1. Department of Mechanical and Process Engineering, The Sirindhorn International Thai-German Graduate School of Engineering (TGGS), King Mongkut’s University of Technology North Bangkok, 1518 Wongsawang Road, Bangsue, Bangkok 10800, Thailand

2. Government Women’s Polytechnic College, Calicut, Kerala, India

*Corresponding authors:

Suchart Siengchin, Email: [suchart.s.pe@tggs-bangkok.org](mailto:suchart.s.pe@tggs-bangkok.org)

Sabarish Radoor, Email: [sabarishchem@gmail.com](mailto:sabarishchem@gmail.com)

Supplementary information (S1)





**Figure S1:** FTIR spectra of ZSM-5 zeolite and PVA/SA/ZSM-5 zeolite membrane
